# Loss of Ercc1 Results in a Time- and Dose-Dependent Reduction of Proliferating Early Hematopoietic Progenitors

**DOI:** 10.1155/2012/783068

**Published:** 2012-06-03

**Authors:** Judith H. E. Verhagen-Oldenampsen, Jurgen R. Haanstra, Paulina M. H. van Strien, Marijke Valkhof, Ivo P. Touw, Marieke von Lindern

**Affiliations:** ^1^Department of Hematology, Erasmus Medical Center, Dr Molewaterplein 50, 3015 GE Rotterdam, The Netherlands; ^2^Department of Hematopoiesis, Sanquin Research and Landsteiner Laboratory, AMC/UvA, Plesmanlaan 125, 1066 CX Amsterdam, The Netherlands

## Abstract

The endonuclease complex Ercc1/Xpf is involved in interstrand crosslink repair and functions downstream of the Fanconi pathway. Loss of Ercc1 causes hematopoietic defects similar to those seen in Fanconi Anemia. *Ercc1* ^−/−^ mice die 3-4 weeks after birth, which prevents long-term follow up of the hematopoietic compartment. We used alternative Ercc1 mouse models to examine the effect of low or absent Ercc1 activity on hematopoiesis. Tie2-Cre-driven deletion of a floxed *Ercc1* allele was efficient (>80%) in fetal liver hematopoietic cells. Hematopoietic stem and progenitor cells (HSPCs) with a deleted allele were maintained in mice up to 1 year of age when harboring a wt allele, but were progressively outcompeted when the deleted allele was combined with a knockout allele. Mice with a minimal Ercc1 activity expressed by 1 or 2 hypomorphic *Ercc1* alleles have an extended life expectancy, which allows analysis of HSPCs at 10 and 20 weeks of age. The HSPC compartment was affected in all Ercc1-deficient models. Actively proliferating multipotent progenitors were most affected as were myeloid and erythroid clonogenic progenitors. In conclusion, lack of Ercc1 results in a severe competitive disadvantage of HSPCs and is most deleterious in proliferating progenitor cells.

## 1. Introduction

The Ercc1/Xpf complex is an endonuclease involved in nucleotide excision repair (NER) and in repair of interstrand crosslinks (ICL) [1, 2]. Mice lacking Ercc1 (*Ercc1^−/−^*) suffer from severe premature aging, which shows as small size, ruffled fur, liver polyploidy, and loss of hematopoietic progenitors from bone marrow (BM), resulting in death at 3-4 weeks of age [3–6]. Hypomorphic *Ercc1* (*Ercc1^d/d^* or Ercc1^∗292^) mice that harbor 2 C-terminally truncated alleles are also small but they survive longer (~6 months), probably as a result of their residual DNA repair capacity (~4%) [1, 2]. The hypomorphic allele has a 7 amino acid deletion at the C-terminus, which impairs dimerization with Xpf [1].

The short life span and severe aging phenotype of *Ercc1^−/−^* is shared with other models of defective NER such as the *Xpa^−/−^ Csb^m/m^* mice that die at 3 weeks of age [7–9]. The hematopoietic defect of *Ercc1^−/−^* mice, however, is specifically linked to defective ICL repair ([5]; Verhagen-Oldenampsen et al., unpublished). The correlation of specific phenotypes with either NER or ICL repair is likely due to the activation of distinct tumor suppressor mechanisms that impact differently on specific tissues. For instance, persistent DNA damage due to defective NER results in deregulation of the growth axis and is independent of p53 and p16^INK4a^  [8]. Hematopoiesis, on the other hand, is particularly sensitive to activation of p53 (Haanstra, Verhagen-Oldenampsen in preparation).

Both fibroblasts and hematopoietic cells of *Ercc1^−/−^* mice and mice lacking Fanconi proteins are hypersensitive to the DNA crosslinker mitomycin C (MMC) [1, 5, 10]. Importantly, the endonuclease complex Ercc1/Xpf participates in the same ICL repair pathway as the Fanconi Anemia (FA) proteins [11, 12]. It associates with FancP/Sxl4 and is required for FancD2 focus formation [13, 14]. Mice lacking for instance the *Fancc* gene only develop hematopoietic defects when challenged with MMC, or when hematopoietic cells are cultured at atmospheric oxygen prior to transplantation [10, 15]. Mice lacking Ercc1 develop hypoplasia of the BM compartment without applying an external challenge similar to FA patients [16] and Fancp/Slx4-deficient mice [14].

The Ercc1 mice are a useful model to study BM failure in FA, which is, however, limited by the short life span of *Ercc1^−/−^* mice. The BM of *Ercc1^−/−^* mice contains fewer progenitors, and the remaining myeloid and erythroid progenitors fail to proliferate *in vitro* [5]. The aim of this study was to characterize progression of BM failure in Ercc1 models with an extended life span, and to examine how low levels of Ercc1 activity impact on hematopoiesis. We used mice with a single floxed *Ercc1* allele and a Tie2-driven Cre recombinase. Tie2 is expressed in the early hematopoietic stem cell (HSC) when they dissociate from the hemogenic endothelium, and in quiescent adult HSC [17, 18]. We show that the *Ercc1* allele recombines efficiently in fetal liver. In presence of an intact *Ercc1* allele, the recombination frequency remained stable, while the frequency of cells lacking *Ercc1* rapidly decreased in BM when the second *Ercc1* allele was lacking. This indicated that Ercc1-deficient hematopoietic cells have a severe competitive disadvantage. To investigate how low levels of Ercc1 affect hematopoietic stem and progenitor cells, we compared hematopoiesis in mice harboring one or two hypomorph alleles (*Ercc1^−/d^* and *Ercc1^d/d^*, encoding proteins with impaired Xpf dimerisation capacity) at 3, 10, and 20 weeks of age. At week 3, we included *Ercc1^−/−^* in this comparison. This analysis showed that proliferating stem and progenitor cells decreased, whereas the most immature cells within the LSK fraction were less affected once these cells became quiescent after 3 weeks of age. The decrease of multipotent progenitors preceded the decrease of committed progenitors indicating that the earliest proliferating progenitors are most sensitive to defective ICL repair.

## 2. Materials and Methods

### 2.1. Animals

*Ercc1^+/d^*, *Ercc1^+/−^* [1], *Ercc1^+/f^* (obtained from Dr. L. Niedernhofer, University of Pittsburgh School of Medicine, Pittsburgh, PA), *Tie2-Cre* [19], and wt littermates were kept in a pure background of both C57/Bl6 and FVB/n at the Animal Resource Center (Erasmus MC). Experimental animals were generated as F1 in a mixed background of C57/Bl6 and FVB/n. *Ercc1^+/−^* and *Ercc1^+/d^* mice displayed a wild-type phenotype and were used as controls. All animal studies were approved by an independent Animal Ethical Committee. Mice were sacrificed by CO_2_ inhalation between postnatal weeks 3 and 20. Neonatal mice and embryo's were sacrificed by decapitation on ice. Femurs, tibia, and sternum were isolated and BM cell suspensions were obtained by crushing the bones in HBSS supplemented with 5% (v/v) foetal calf serum, 100 units/mL penicillin, and 100 *μ*g/mL streptomycin. Fetal livers and neonatal spleens were resuspended by pipetting in the same medium.

### 2.2. Colony-Forming Unit Assays

Bone marrow cell suspensions were plated in methyl cellulose medium (Methocult M3234, StemCell Technologies SARL, Grenoble, France) containing huGCSF (0.1 *μ*g/mL), muGM-CSF (0.1 *μ*g/mL), or Epo (4 mU/mL) plus transferrin (0.3 mM), hemin (0.2 mM), and muSCF (0.1 *μ*g/mL). Colonies containing 30 cells or more were scored after 7-8 days of culture.

### 2.3. Flow Cytometry

Single-bone-marrow cell suspensions were analyzed by flow cytometry using a BD LSR II Flow Cytometer System with FCS Express Diva software (BD Biosciences, San Jose, CA). FCS files were analyzed using FlowJo (Tree Star, Inc., Ashland, OR). Cells were labelled with the following antibodies; mouse biotinylated lineage depletion kit, CD16/CD32-PE, CD117-APC, CD135-PE and streptavidin-APC-Cy7 (BD Pharmingen), Sca1-PE-Cy7, CD34-pacific blue and CD127-pacific blue (Ebioscience), and 7′AAD (Invitrogen).

### 2.4. Genotyping PCR and Q-PCR

Genomic DNA was isolated from tail segments or from blood (NucleoSpin Tissue XS, MACHEREY-NAGEL GmbH & Co). Genotypes were determined by PCR. Genomic Q-PCR used an Applied Biosystems 7900 instrument (Applied Biosystems, Weiterstadt, Germany) and SYBR Green PCR Master Mix (Applied Biosystems). Primers used were HPRT—forward: AGCCTAAGATGAGCGCAAGT, reverse: ATGGCCACAGGACTAGAACA; Recombined Ercc1 allele—forward: TGCAGCATGCTCTAGACTCG, reverse: CCATGAATTCCGGGATCTCTCGAC; nonrecombined Ercc1 allele—forward: TCCACTTCGCATATTAAGGTGA, reverse: AACCTGCGTGCAATCCAT; Ercc1 knock out locus—forward: TCCTCGTGCTTTACGGTATC, reverse: CAGGATCAGGAGGTACAGGA.

### 2.5. Histology

Livers were embedded in Tissue-Tek O.C.T (Sakura Finetek, Zoeterwoude, Netherlands). 4 *μ*m sections were made using a cryostat (Leica) and stained with hematoxylin and eosin. Slides were imaged on a Leica DMLB light microscope equipped with Leica application suite 2.7.1 (Leica Microsystems, Switzerland).

## 3. Results

### 3.1. Ercc1-Deficient Hematopoietic Stem and Progenitor Cells Have a Competitive Disadvantage

**Figure 1:**
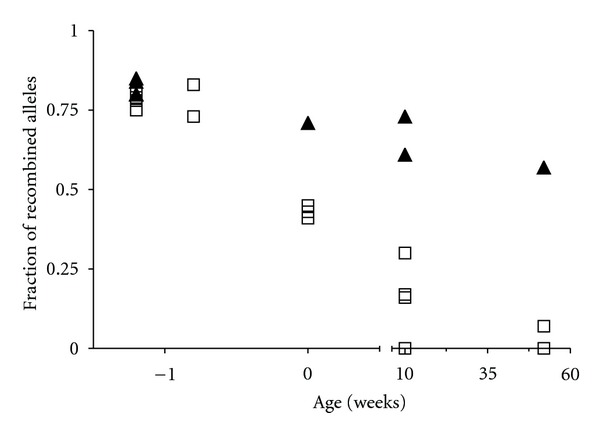
Recombination in Ercc1-flox Tie2-Cre model. The fraction of recombined alleles in the presence of Tie2-Cre was calculated after measuring the nondeleted floxed allele by real-time genomic PCR and comparing it to the presence of the floxed allele in absence of Cre. HPRT was measured to control for total DNA. DNA was isolated from fetal livers at embryonic days E12.5 and E15.5, from the spleen of neonatal mice, and from bone marrow of 10- and 52-week-old mice. Closed triangles: *Tie2-Cre; Ercc1^+/f^*, open boxes: *Tie2-Cre; Ercc1^−/f^*. Each symbol is an independent measurement.

*Ercc1^−/−^* mice have an average lifespan of 3 weeks. Because we aimed to study long-term effects of Ercc1-deficiency on hematopoietic stem cell function, we used a Cre-lox conditional mouse model expressing Cre-recombinase from the Tie2 promoter (*Tie2-Cre*). Tie2 is expressed on vascular endothelial cells and HSCs [17, 18]. Mice with a single floxed Ercc1 allele (*Ercc1^+/f^*) were crossed with *Ercc1^+/−^ Tie2-Cre* mice. We compared *Ercc1^−/f^* and *Ercc1^+/f^* mice with and without expression of Tie2-Cre. Because the recombination efficiency in Cre-lox mouse models is never 100% [20], deletion of the floxed allele was analyzed both pre- and postnatal in the most active hematopoietic organ, that is, fetal liver in the embryo, spleen in newborn animals, and BM in adult animals. The presence of the floxed allele was analyzed by real-time genomic PCR on DNA isolated from the various tissues. The fraction of cells with a deleted floxed allele was calculated by comparing the relative signals in tissues with or without Cre. *Tie2-Cre/Ercc1^+/f^* mice showed stable deletion of the floxed allele in 50% or more of the hematopoietic cells (Figure 1). In *Tie2-Cre/Ercc1^−/f^* mice, the Ercc1 allele was deleted in 80% of fetal liver cells at prenatal days E12.5 and E15.5. In newborn *Tie2-Cre/Ercc1^−/f^* animals (postnatal day 1), ~50% of spleen cells carried a deleted floxed allele. At ten weeks of age, the recombined allele was undetectable or present in a low percentage of cells. In *Tie2-Cre/Ercc1^−/f^* animals of 1 year old, the BM contained hardly any cells with a recombined allele (Figure 1). Accordingly, blood cell parameters and colony-forming progenitors in BM were similar in *Ercc1^−/f^* mice with or without *Tie2-Cre* expression at 10 weeks and 1 year of age (data not shown). This indicates that *Ercc1-*deleted cells are outcompeted by cells in which the floxed allele was not recombined. The presence of one *Ercc1* allele is sufficient to maintain the hematopoietic cell compartment at a similar level as in nondeleted animals.

### 3.2. The Composition of the Hematopoietic Stem Cell Pool Is Affected by the Level of Ercc1 Activity

**Figure 2:**
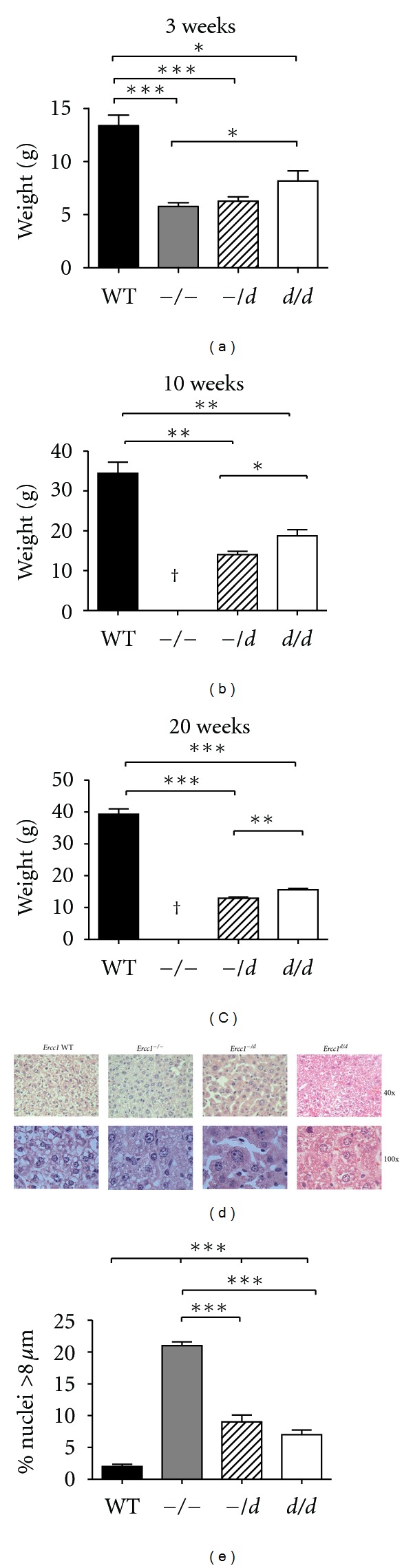
Weight and liver cell morphology of mice with distinct levels of Ercc1 activity. ((a)–(c)) Mean body weight of (a) 3-week-old *Ercc1^−/−^* (*n* = 6), *Ercc1^−/d^* (*n* = 7), *Ercc1^d/d^* (*n* = 4), and wt (*n* = 13) mice. (b) 10-week-old *Ercc1^d/d^* (*n* = 3), *Ercc1^−/d^* (*n* = 3), and wt (*n* = 6) mice. (c) 20-week-old *Ercc1^-/d^* (*n* = 8), *Ercc1^d/d^* (*n* = 5), and wt (*n* = 12) mice. (d) Hematoxylin- and eosin-stained sections of liver from 3-week-old wt, *Ercc1^−/−^*, *Ercc1^−/d^*, and *Ercc1^d/d^* mice. (e) Quantification of enlarged nuclei (>8 *μ*m). Error bars indicate standard deviation. *indicates *P* ≤ 0.05, **indicates *P* ≤ 0.01, and ***indicates *P* ≤ 0.001.

To find a window of Ercc1 expression that allows for the analysis of hematopoiesis for several weeks, we compared hematopoiesis in bone marrow of *Ercc1^−/−^* mice with mice harboring one C-terminally truncated Ercc1 allele and a knock out allele (*Ercc1^−/d^*), or two C-terminally truncated Ercc1 alleles (*Ercc1^d/d^*). The truncated allele has been described as *293 [1] or as *delta* [21], we adopted *delta*, indicated as “*d*”, that should not to be confused with a recombined floxed allele. Three-week-old mice with low or absent Ercc1 activity had a dose-dependent decrease in body size (Figure 2(a)). *Ercc1^−/−^* mice died between weeks 3 and 4. The *Ercc1^d/d^* and *Ercc1^−/d^* mice survived longer, but their low body weight persisted at 10 and 20 weeks of age (Figures 2(b) and 2(c)). A comparison of liver morphology of the various Ercc1-deficient mice at 3 weeks of age indicated that livers from both *Ercc1^−/−^, Ercc1^−/d^, *and *Ercc1^d/d^* mice contained cells with enlarged nuclei, compared to wt livers (larger than 8 *μ*m; Figures 2(d) and 2(e)) as previously described [21].

To analyze the effect of low levels of Ercc1 on hematopoiesis, we first examined the stem and progenitor cell compartment using flow cytometry. Hematopoietic stem cells and progenitor cells (HSPCs) were defined as negative for lineage markers (Lin−) and positive for the surface markers Sca (Sca1+) and the SCF receptor cKit (cKit+), indicated as the LSK fraction. The stem cell compartment was further subdivided into long-term HSC (LT-HSC, CD34−, CD135−), short-term HSC (ST-HSC, CD34+ CD135−), and multipotent progenitors (MPP, CD34+ CD135+) [22].

**Figure 3:**
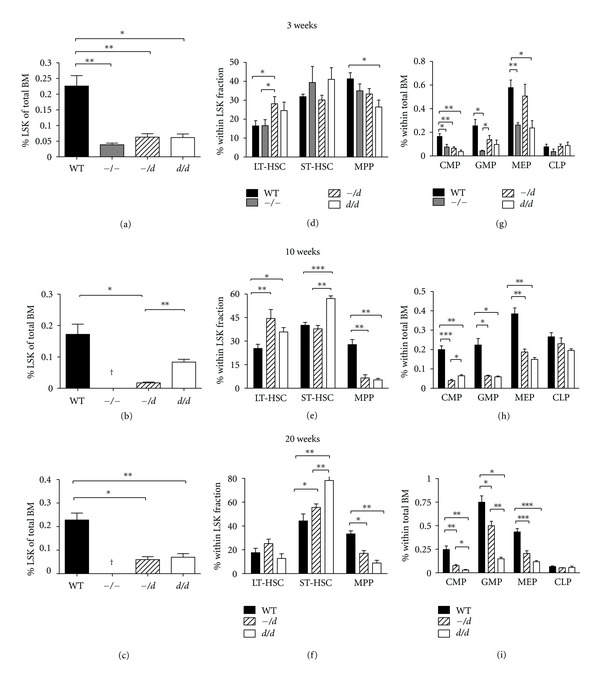
Ercc1 levels influence the composition of the stem and progenitor cell pool. Whole BM suspensions were stained with surface antigen specific antibodies for hematopoietic stem cells. ((a)–(c)) LSK (Lin− Sca1+ cKit+) cells as percentage of total bone marrow cells. ((d)–(f)) Distribution of stem cells within the LSK fraction (LT HSC(CD34− CD135−), ST-HSC (CD34+ CD135−) and MPP (CD34+ CD135+)). ((g)–(i)) Distribution of progenitor cells within total bone marrow (CMP (Lin− cKit+ CD34+ CD16/CD32intermediate), GMP (Lin− cKit+ CD34+ CD16/CD32high), MEP (Lin− cKit+ CD34− CD16/CD32low) and CLP (Lin− CD127+ Sca1/cKit intermediate)). Mean percentages are plotted; error bars indicate standard deviation. *indicates *P* ≤ 0.05, **indicates *P* ≤ 0.01 and ***indicates *P* ≤ 0.001.

Because BM cellularity corrected for body weight was comparable between the different genotypes at 3, 10, and 20 weeks of age, a comparison of the subset ratios was permitted between Ercc1-deficient mice and their wt littermates. The percentage of LSK cells in the total bone marrow of 3-week-old mice was decreased to 17% of wt for *Ercc1^−/−^*, 28% of wt for *Ercc1^−/d^*, and 27% of wt in *Ercc1^d/d^* mice (Figure 3(a)). At 10 weeks of age, the percentage of LSK cells present in the BM further decreased in *Ercc1^−/d^* mice to 10% of wt but stabilized to 50% of wt for *Ercc1^d/d^* mice (Figure 3(b)). At 20 weeks, the percentage of LSK was 26% of wt for *Ercc1^−/d^* and 31% of wt for *Ercc1^d/d^* (Figure 3(c)). Thus, the size of the stem cell compartment correlates with Ercc1 activity but fluctuates over time.

We next investigated how distinct subpopulations within the LSK compartment depend on Ercc1 protein activity. The distribution of LT-HSC, ST-HSC, and MPP displayed relatively minor changes at week 3 (Figure 3(d)). At 10 weeks of age, the fraction of actively dividing MPP was more than 3-fold decreased in both hypomorphic models (Figure 3(e)). The *Ercc1^−/d^* BM contained predominantly quiescent LT-HSC, while proliferating ST-HSC was the most abundant fraction in *Ercc1^d/d^* BM (Figure 3(e)). The enrichment of quiescent LT-HSC is in accordance with the further reduction of LSK in *Ercc1^−/d^* BM. In contrast, the LSK fraction in *Ercc1^d/d^* BM partly recovered at week 10, which is in accordance to the increase in ST-HSC fraction. At 20 weeks of age, the distribution of quiescent and dividing subfractions within the population of LSK cells remained similar to the distribution at 10 weeks for both *Ercc1^−/d^* and *Ercc1^d/d^* mice (Figure 3(f)).

To specify the distribution of progenitors that arise from the LSK fraction in relation to the remaining Ercc1 activity, we analyzed the following lineage committed progenitor subsets: common myeloid progenitors (CMP, Lin− cKit+ CD34+ CD16/CD32int), granulocyte-monocyte progenitors (GMP, Lin− cKit+ CD34+ CD16/CD32hi), megakaryocyte-erythroid progenitors (MEP, Lin− cKit+ CD34− CD16/CD32low), and common lymphoid progenitors (CLP, Lin− CD127+ Sca1/cKit+ int). At 3 weeks of age, the CMP fraction of *Ercc1^−/−^* mice decreased to 46% of wt, the GMP fraction to 16% of wt, the MEP fraction to 45% of wt, and the CLP fraction to 48% of wt levels (Figure 3(g)). In *Ercc1^−/d^* mice, the progenitor subsets decreased to, respectively, 39%, 54%, and 88% of wt levels and no change in CLP levels (Figure 3(g)). For *Ercc1^d/d^* mice, these percentages were 23%, 38%, and 41% of wt levels and no difference in CLP levels (Figure 3(g)). For all myeloid subsets, except the CMP compartment, the numbers increased in *Ercc1^−/d^* mice as compared to *Ercc1^−/−^* mice.

At 10 weeks of age, BM of *Ercc1^−/d^* mice contained 20% of wt CMP levels, 29% of wt GMP levels, 49% of wt MEP levels, and 87% of wt CLP levels (Figure 3(h)). In *Ercc1^d/d^* mice, these subsets contained 32%, 27%, 39%, and 74% of wt levels, respectively (Figure 3(h)). At 20 weeks of age, *Ercc1^−/d^* BM contained 35% of wt CMP levels, 77% of wt GMP levels, 53% of wt MEP levels, and 67% of wt CLP levels (Figure 3(i)). In *Ercc1^d/d^* mice, these subsets contained 13%, 23%, 31%, and 69% of wt levels, respectively (Figure 3(i)).

In conclusion, decreased Ercc1 levels reduce all compartments of actively proliferating stem and progenitor cells except for the CLP fraction that is only moderately affected. Despite reduced numbers of progenitors in BM, we observed normal cell numbers in peripheral blood (data not shown). The presence of a hypomorphic Ercc1 allele extends the life span of the mice and marginally improves hematopoiesis in the mice. Also in *Ercc1^d/d^* mice, the number of HSPCs remains severely compromised.

### 3.3. Ercc1 Deficiency Impairs Colony Formation by Hematopoietic Progenitors

**Figure 4:**
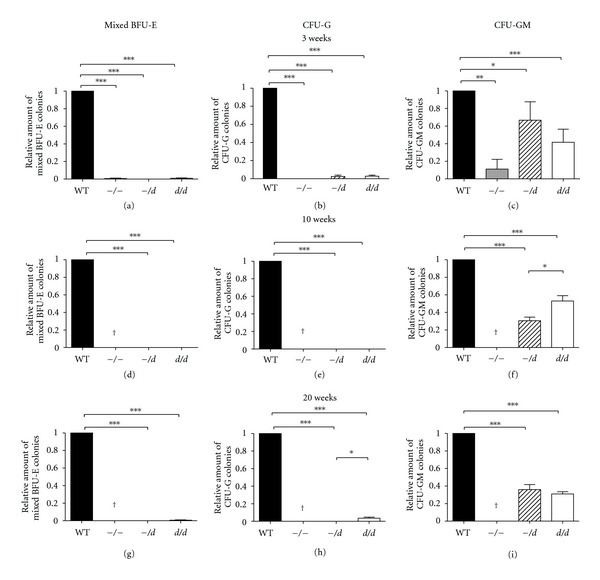
Colony-forming potential of bone marrow progenitors is affected in hypomorphic models or Ercc1. BFU-E, CFU-G, and CFU-GM colonies per 5 × 104 bone marrow cells derived from ((a)–(c)) 3-week-old *Ercc1^−/−^* (*n* = 6), *Ercc1^−/d^* (*n* = 7), *Ercc1^d/d^* (*n* = 4), and wt mice (*n* = 7) ((d)–(f)) 10-week-old *Ercc1^−/d^* (*n* = 3), *Ercc1^d/d^* (*n* = 3), and wt mice (*n* = 3), ((g)–(i)) 20-week-old *Ercc1^−/d^* (*n* = 3), *Ercc1^d/d^* (*n* = 6), and wt mice (*n* = 8). Error bars indicate standard deviation. *indicates *P* ≤ 0.05, **indicates *P* ≤ 0.01, and ***indicates *P* ≤ 0.001.

To assess the colony-forming potential of hematopoietic progenitors, bone marrow suspensions were plated in semisolid medium supplemented with lineage-specific cytokines. At 3 weeks of age, the number of erythroid (BFU-E, Figure 4(a)), granulocytic (CFU-G, Figure 4(b)), and granulocytic-macrophage colony-forming cells (CFU-GM, Figure 4(c)) were significantly reduced in all Ercc1-deficient models relative to wt (Figures 4(a)–4(c)).

Similar results were obtained in BM of 10- and 20-week-old mice; *Ercc1^−/d^* BM formed no BFU-E colonies (Figures 4(d) and 4(g)), no CFU-G colonies (Figures 4(e) and 4(h)) and only 31% of CFU-GM colonies compared to wt (Figures 4(f) and 4(i)). In *Ercc1^d/d^* BM, the percentages were 0%, 0%, and 35% of wt, respectively. These results imply that the residual Ercc1 activity in *Ercc1^−/d^* and *Ercc1^d/d^* mice is not sufficient to support BFU-E or CFU-G colony formation, whereas CFU-GM colony outgrowth is only partly restored.

## 4. Discussion

The Ercc1/Xpf endonuclease complex acts downstream of the Fanconi pathway in ICL repair [1, 2, 12]. The hematopoietic defects in Ercc1-deficient mice are reminiscent of the hematopoietic defect of FA patients [23]. It mostly takes several years before FA patients develop anemia. In most FA mouse models, loss of HSC is only seen when the mice are challenged with Mitomycin C [24, 25]. The fact that most mouse models lacking Fanconi genes fail to display overt BM failure may reflect the time it takes to develop anemia. An important factor in the onset of BM failure and leukemia development may be the level of residual DNA repair activity. We employed Ercc1-deficient mouse models to show progressive loss of the number of hematopoietic stem and progenitor cells dependent on Ercc1 activity. Remaining progenitors were compromised in their *in vitro* proliferation capacity, which was similarly severe in *Ercc1^−/−^*, *Ercc1^−/d^*, and *Ercc1^d/d^* mice.

### 4.1. Reduced Competitiveness of Ercc1-Deficient Hematopoietic Cells

The conditional knock out model showed that a small percentage of hematopoietic stem and progenitor cells in which the floxed Ercc1 allele did not recombine outcompeted the Ercc1-deficient cells in which Cre-driven deletion had occurred. This progressive loss of Ercc1-deficient hematopoietic cells resembles what has been found in a small fraction of FA patients. In some FA patients, a mutation was reverted because two mutated alleles were recombined and yielded an unaffected allele. Such a naturally corrected hematopoietic stem cell is able to out-compete the hematopoietic cells with two defective alleles resulting in the restoration of BM cellularity. In these patients, the fibroblasts retained two mutated alleles [26]. The conditional knock out mice that we used here underscore that defective ICL repair mainly affects continuously regenerating tissues such as the hematopoietic system. It is also in the continuously proliferating bone marrow compartment that few cells with an intact allele can out-compete cells that lack a functional FA pathway.

### 4.2. Reduced Hematopoietic Reserves with Normal Peripheral Blood Levels

The hematopoietic defect in Ercc1-deficient mice, and in FA, is specifically associated with DNA crosslinks that stall the replication fork. The inability to repair spontaneous DNA damage limits stress-hematopoiesis by diminishing the ability of HSCs to proliferate and self-renew. During embryo development and in young mice (<3 weeks), the HSC compartment is continuously expanded, whereas HSC become largely quiescent in adult mice [27, 28]. These quiescent HSCs are less sensitive to replication-coupled DNA damage repair defects. Progenitor cells have a higher proliferation rate compared to HSC and are, therefore, more prone to DNA damage both during development and in adult mice. Accordingly, we found that LSK numbers are 3- to 5-fold decreased compared to their wt littermates in *Ercc1*-deficient mice. At 3 weeks of age, the distribution within the LSK compartment hardly shows a tendency towards more primitive cells, most likely because all compartments contained proliferative cells. At 10 and 20 weeks of age, when the mice are adult, there is a significant shift towards the more primitive cells in the LSK compartment in the *Ercc1^−/d^* and *Ercc1^d/d^* compared to their wt littermates, indicating that maintenance of the LT-HSC fraction is less sensitive to DNA interstrand cross links than the maintenance of the proliferative MPP fraction [29].

 However, the mice did not develop overt anemia, and peripheral blood contained near normal amounts of red and white blood cells. This is most likely due to compensatory mechanisms controlled by a network of cytokines and hormones: only small and transient alterations in local- and/or systemic concentrations will be needed to maintain or restore homeostasis. Notably, Epo serum concentrations were normal in Ercc1-deficient mice (data not shown), but this result was expected given that the mice were not anemic and Epo production in the kidney is activated by hypoxia.

Because cell numbers in peripheral blood are hardly affected, the hematopoietic defect in *Ercc1^−/d^* mice does not represent overt BM failure but can be regarded as a situation prone to such overt BM failure. Also in FA patients, reduced stem cell numbers precede overt BM failure and leukemia development [30, 31]. When challenged for regeneration following insult, the Ercc1-deficient stem and progenitor cells lack the robustness to do so. Analysis of BM and leukemogenesis in FA and in FA mouse models shows that hypoplasia precedes leukemic transformation [32, 33]. Hypoplastic compartments are most at risk for leukemic transformation [34]. FA patients mainly develop acute myeloid leukemia (AML) and only very rarely acute lymphoid leukemia (ALL) [35]. In the Ercc1 models, we also found that the myeloid compartment is affected by Ercc1 deficiency while the CLP compartment is hardly affected. Therefore, the hypomorphic Ercc1 mice may be a very useful model to study BM failure mechanisms and subsequent leukemogenic transformation in FA.

### 4.3. Comparison of the Hematopoietic Phenotype of *Ercc1^−/−^*, *Ercc1^−/d^*, and *Ercc1^d/d^* Mice

In myeloid and erythroid colony-forming assays, *Ercc1*-deficient progenitors show a 50% (on GM-CSF) to a 100% (on EPO/SCF or G-CSF) decrease in colony numbers. In *Ercc1^−/d^*, and *Ercc1^d/d^* mice, the decrease in colony numbers was not significantly different from those in *Ercc1^−/−^* mice. This implies that low levels of functional protein cannot repair the damage inflicted by the rapid proliferation that occurs in these assays. Flow cytometry measurements indicated that the decrease in myeloid and erythroid colony-forming cell numbers was only moderate in the Ercc1-deficient models at 3 weeks of age. Thus, the progenitors are present, and they are able to generate progeny *in vivo*, but not *in vitro*. *In vitro* conditions challenge the proliferation capacity more than the *in vivo* condition and may be more mutagenic such as higher oxygen levels.

##  Conflict of Interests

The authors have no conflict of interests.

##  Authors' Contributions

J. H. E. Verhagen-Oldenampsen and J. R. Haanstra contributed equally to this paper. I. P. Touw and M. von Lindern share equal responsibility of this paper.
